# A65 THE YAP-PER: CAN YAP/TAZ MEDIATE EPITHELIAL-IMMUNE CROSSTALK DURING PYLORIC METAPLASIA?

**DOI:** 10.1093/jcag/gwad061.065

**Published:** 2024-02-14

**Authors:** J Sung, A Gregorieff, S Gruenheid

**Affiliations:** Microbiology and Immunology, McGill University, Montreal, QC, Canada; Microbiology and Immunology, McGill University, Montreal, QC, Canada; Microbiology and Immunology, McGill University, Montreal, QC, Canada

## Abstract

**Background:**

Gastric cancer is one of the leading causes of cancer-related deaths and its prevalence has been associated with infection by *Helicobacter pylori*. *H. pylori* persistence leads to chronic inflammation, which results in the elimination of acid-secreting parietal cells and the appearance of abnormal mucus-producing cells at the base of the gastric glands, known as spasmolytic polypeptide/trefoil factor 2-expressing metaplasia (SPEM) cells. The processes driving these alterations in epithelial cell fate during *H. pylori* infection and their function in regeneration of the gastric epithelium remain unclear. Previous work done in our lab have demonstrated that Hippo signalling, more specifically the transcriptional effectors yes-associated protein 1 (YAP) and transcription coactivator with PDZ-binding motif (TAZ), plays an essential role in controlling cell fate during gastric tissue regeneration in a chemical injury model. In the same model, loss of YAP/TAZ resulted in chronic immune cell infiltration in corpus glands, suggesting an immunomodulatory role for YAP and TAZ.

**Aims:**

The objectives of this project is to characterize the immunomodulatory function of YAP/TAZ in an *in vivo* model of acute injury as well as an *in vivo* model of *H. pylori* infection.

**Methods:**

Acute injury is induced by high-dose tamoxifen treatment in conditional knock-out (cKO) mice with YAP/TAZ deleted throughout the gastric epithelium. Subsequent immunophenotyping experiments such as flow cytometry and multiplex analysis are performed to characterize the role played by YAP/TAZ in regulating the immune response. For a more biologically relevant model, cKO mice are also infected with *H. pylori,* and similar immunophenotyping analyses are performed.

**Results:**

Preliminary results showed that the loss of YAP/TAZ resulted in differential type II response as well as chronic B cell infiltration upon tissue injury.

**Conclusions:**

This project aims to provide important insights on immune-epithelial cell interactions important for tissue regeneration and tumorigenesis as well as host-pathogen interactions implicated in *H. pylori* persistence.

**Funding Agencies:**

CIHRFRQS

